# Dual diagnosis of bipolar disorder and substance use disorder – type of substance used and its impact on treatment adherence and maintenance of abstinence

**DOI:** 10.1192/j.eurpsy.2024.750

**Published:** 2024-08-27

**Authors:** I. A. Silva, C. Silva, I. Faria, V. S. Melo

**Affiliations:** ^1^Unidade Local de Saúde do Norte Alentejano, Portalegre; ^2^Centro Hospitalar e Universitário de Coimbra, Coimbra; ^3^Centro Hospitalar do Médio Tejo, Tomar, Portugal

## Abstract

**Introduction:**

Substance use disorder is a common comorbidity with bipolar disorder, delaying its diagnosis and making treatment of both disorders more complex and challenging.

**Objectives:**

We aim to analyze the types of substances used by patients with bipolar disorder and to find if there’s a relationship between the substance used both with treatment adherence and maintenance of abstinence.

**Methods:**

We collected, retrospectively, data from the hospital platform and analyzed it on SPSS Statistics 26, along with a literature review. Our study looks over 3 years, and all patients analyzed have a dual diagnosis of both bipolar disorder and substance use disorder and were hospitalized in the psychiatric ward of a tertiary university hospital.

**Results:**

There were 2384 hospitalizations in the Coimbra’s University Hospital psychiatric ward, and 88 hospitalizations were coded with a dual diagnosis of bipolar disorder and substance use disorder.

Tobacco was the substance more consumed by the patients (53.4%), followed by alcohol (46.6%) and cannabinoids (30.7%). In 18.2% of the patients was identified consumption of cocaine and in 6.8% there was an abuse of opioids. It is important to highlight that 20.5% of the patients used 2 or more substances at the same time.

Regarding adherence to treatment for both their bipolar disorder and substance use disorder, in 25% of the patients, there wasn’t a satisfactory compliance with the treatment prescribed.

In the group of patients with polydrug use, half of them didn’t comply with the treatment. In the patients consuming only one substance, we found out that 30% of patients who use alcohol didn’t adhere to the treatment, while around 13% of the patients using cannabinoids didn’t comply with the suggested treatment.

The relationship between the type of substance used and treatment adherence was statistically significant with a p=0.004 (considering p<0.05).

Regarding abstinence from consumption, around 42% of the patients keep using at least one substance. In the group with polydrug use, around 65% of the patients were not abstinent in the last appointments, while in the cannabinoids users’ group around 50% of them were still using the drug. In the group with patients using alcohol, around 43% of them are not abstinent.

The relationship between the type of substance used and maintenance of abstinence was found to be statistically significant with a p=0.037 (considering p<0.05).

**Conclusions:**

Substance use disorder can have a huge impact on adherence to treatment, worsening the prognosis of the comorbid bipolar disorder. On the other hand, this dual diagnosis can impact the maintenance of abstinence.

Early detection of both diagnosis and simultaneous treatment from an early phase are essential to improve the prognosis of both diseases.

**Disclosure of Interest:**

None Declared

